# Preclinical Optimization of Magnetotactic Bacteria Therapy for the Treatment of Pancreatic and Rectal Cancer ^†^

**DOI:** 10.3390/cancers18152427

**Published:** 2026-07-28

**Authors:** Charles Tremblay, Miriam Santos Dutra, Gilles Soulez, Sylvain Martel, Gerald Batist, Corey S. Miller

**Affiliations:** 1 NanoRobotics Laboratory, Department of Computer and Software Engineering, Institute of Biomedical Engineering, Polytechnique Montréal, Montreal, QC H3T 1J4, Canada; ctremblay@polymtl.ca (C.T.);; 2 McGill Centre for Translational Research in Cancer, Lady Davis Institute for Medical Research, McGill University, Montreal, QC H3T 1E2, Canada; 3 Department of Radiology and Nuclear Medicine, Centre Hospitalier de l’Université de Montréal (CHUM), Montreal, QC H2X 0C1, Canada; 4 Segal Cancer Centre, Jewish General Hospital, McGill University, Montreal, QC H3T 1E2, Canada; 5 Division of Gastroenterology, Department of Medicine, Jewish General Hospital, McGill University, Montreal, QC H3T 1E2, Canada

**Keywords:** intratumoral, endoscopic ultrasound, ultrasound, cone-beam computed tomography, fiducial markers, image-guided needle navigation, medical imaging, contrast bolus

## Abstract

**Background/Objectives**: Magnetotactic bacteria therapy is an emerging active intratumoral drug delivery platform in which drug-loaded, magnetically responsive bacteria are injected into a tumor and navigated through its microenvironment toward defined targets using an external magnetic field, fundamentally distinct from passive intratumoral injection. No endoscopic ultrasound (EUS)- or ultrasound (US)-guided delivery workflow for MTBT has been described, and five critical technical prerequisites for clinical translation remain unaddressed: three-body image registration, minimum contrast concentration for cone-beam computed tomography (CBCT) bolus localization, needle repositioning accuracy, optimal fiducial strategy, and EUS/US procedural feasibility within the magnetic guidance apparatus. **Methods**: In three healthy female swine, we tested an image-guided intratumoral workflow at pancreatic and rectal sites. Gold rod fiducials were implanted under EUS in two animals and via transabdominal/endorectal US in one animal. A needle was navigated toward the implanted fiducial group using real-time gold-fiducial shine-through, and 2 mL of saline mixed with iodine contrast (Isovue-370) at 15%, 10%, or 5% *v/v* (one concentration per animal) was injected. Scan with CBCT was acquired before and after injection; fiducials, needle tip, and contrast bolus were segmented in 3D Slicer and their centroids compared to quantify targeting accuracy. Contrast visibility and artifact were scored by an expert radiologist on a five-point Likert scale. EUS feasibility within the magnetic apparatus was assessed using a full-scale cardboard replica of the CuraDrone PolarTrak. **Results**: Five percent *v/v* Isovue-370 was the minimum concentration for reliable CBCT bolus identification (280–360 HU; 1:1 injection-to-volume ratio). Needle repositioning accuracy was ≤2 cm in 5/6 sites using fiducial shine-through guidance. Gold rod fiducials served a dual role: CBCT tumor registration and real-time EUS/US navigation, not previously described for intratumoral injection. EUS was feasible within the PolarTrak with defined workflow adaptations. **Conclusions**: This pilot study demonstrates the technical feasibility of an EUS/US–CBCT workflow for MTBT, yielding quantitative, actionable parameters for first-in-human trial design. These feasibility findings, obtained in a small healthy animal cohort, are intended to inform the design of subsequent clinical studies.

## Simple Summary

Magnetotactic bacteria therapy (MTBT) is a new cancer treatment in which drug-loaded, magnetically steerable bacteria are injected into a tumor and actively driven through it, fundamentally different from conventional drug injection. Delivering MTBT to deep targets like the pancreas and rectum requires endoscopic ultrasound or percutaneous ultrasound guidance within a novel magnetic apparatus. This study reports, for the first time, a complete image-guided workflow for MTBT delivery, addressing the key technical prerequisites for first-in-human clinical trial design.

## 1. Introduction

**Figure 1:**
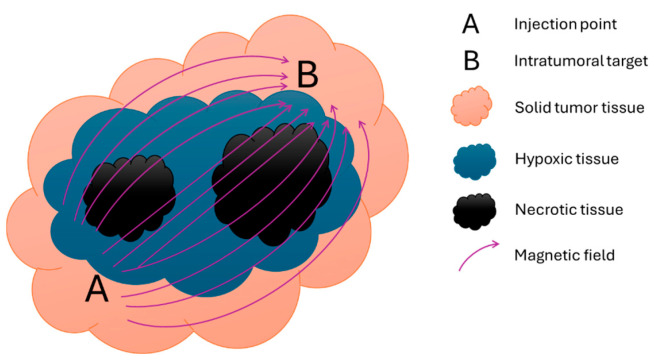
Schematic of the intratumoral MTBT showing the injection point A to target point B used in the magnetic guidance concept for dose distribution using magnetotactic motion of the bacteria. The tumor subregions depicted are viable, hypervascular, hypoxic and necrotic tissues.

Magnetotactic bacteria therapy is a novel cancer treatment that consists of the intratumoral injection of drug-loaded magnetotactic bacteria [1,2]. The therapy (Figure 1) relies on the ability of magnetotactic bacteria (MTB) to migrate along the magnetic field line that induces a directional torque on a chain of nanoparticles in each MTB cell and to distribute the drug inside a tumor lesion from an initial injection point (A-point) toward at least one target (B-point) [3,4].

**Figure 2:**
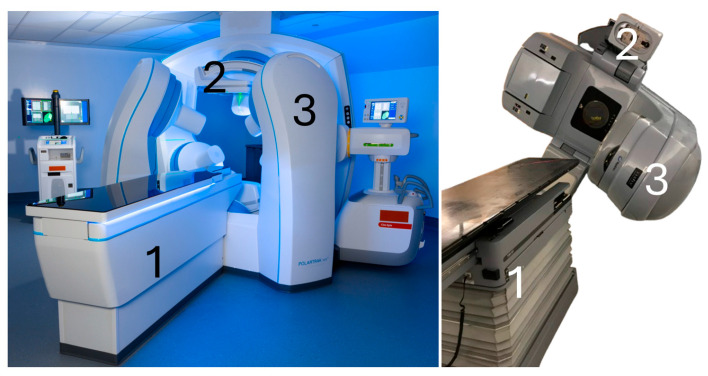
(**Left**) MTBT CuraDrone PolarTrak components (reproduced with permission from CuraDrone, Montréal, QC, Canada); (**Right**) IGRT Varian TrueBeam (reproduced under CC0 1.0). In both images, 1—robotic table, 2—CBCT, 3—radiation sources.

In its current implementation, the treatment apparatus capable of generating the required magnetic fields, the PolarTrak^TM^ (CuraDrone, Montreal, QC, Canada), is equipped with a 3D robotic table and operates in conjunction with an integrated mobile interventional CBCT (Siemens CIOS Spin, Erlangen, Germany) used for tumor registration. The system components (Figure 2) are analogous to those of an image-guided radiotherapy (IGRT) treatment system [5,6].

**Figure 3:**
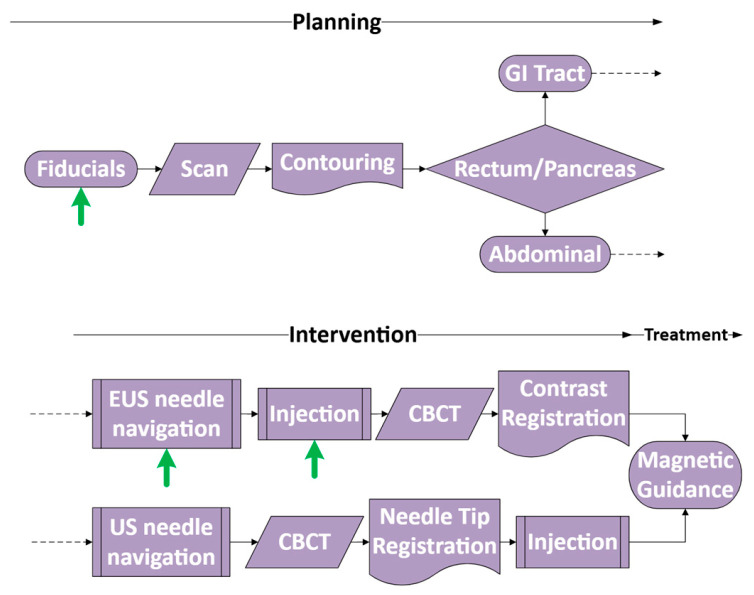
Two MTBT workflows. The green arrows show the EUS steps discussed in this work.

Consequently, the MTBT treatment workflow is inspired by external beam radiotherapy (EBRT), in which the immobilized patient anatomy and target lesion need to be known relative to a radiation-generating apparatus [7,8] to which MTBT adds an intratumoral injection step using US or EUS for needle insertion [9,10,11]. Overall, the three elements: the injected bolus of bacteria, the tumor and the magnetic field must be registered to localize the injected bolus within the tumor, the tumor localization and the contours in order to plan the magnetic guiding sequences enabling adequate distribution of MTBTs in the tumor. Figure 3 shows the overall proposed MTBT workflow and the two alternative routes for needle navigation.

### 1.1. Registration of Tumor Using Fiducials and Fiducial Selection for MTBT

The interventional imaging modality used in IGRT for registration, CBCT, is also chosen for MTBT injection registration. While CBCT can reveal bony anatomy in 3D with high precision, its contrast sensitivity is not sufficient to distinguish soft tissue structure or cancer lesions [12]. Tumor registration in radiation oncology is often performed by using implanted fiducials visible on CBCT and other preoperative imaging modalities placed around the tumor. The fiducial markers are landmarks used to perform the registration and position tumors that are not visible on the CBCT in the 3D space to localize them and plan the radiotherapy [13,14,15]. This is also why the MTBT workflow proposes to use implanted fiducials near the lesion for registration of the otherwise invisible tumor with the integrated PolarTrak CBCT [15,16,17].

Metallic fiducial markers are clearly visible on CBCT and computed tomography (CT) scans, making them reliable tools for tumor localization during treatment planning [18]. Gold is commonly used due to its biocompatibility and visibility on these imaging modalities. However, gold markers are less effective on MRI, where they often appear as signal voids and can sometimes cause artifacts. While certain modifications to the marker’s shape or composition can improve MRI visibility, these changes often introduce new challenges, such as artifacts or handling difficulties. Simple rod-shaped gold fiducials are easier to handle, more cost-effective, and work well with EUS placement [13,15,19,20]. Although they may offer limited MRI visibility, their identification can be confidently confirmed by aligning MRI with CT or CBCT images [21]. For these reasons, MTBT and this study use simple rod-shaped fiducials for tumor marking during the pretreatment planning phase.

### 1.2. Interventional US/EUS

For intratumoral injection, tumors can either be assessed using surface or endo-cavity probe US for tumor sites involving the head and neck, prostate, breast, and skin [22], or by EUS for the gastrointestinal (GI) tract and pancreas. Interventional US is a common technique for general needle navigation in applications including biopsy, fiducial implantation, tumor ablation or catheterization [23,24,25]. This approach can be used for surface lesions and some visceral lesions. EUS is the favored method for examining and accessing the GI system and adjacent organs, including for fiducial placement and biopsy [26]. EUS is an emerging technique for cancer therapy with fine-needle injection (FNI) [24], originally developed for small tumor tattooing before surgery [27].

**Figure 4:**
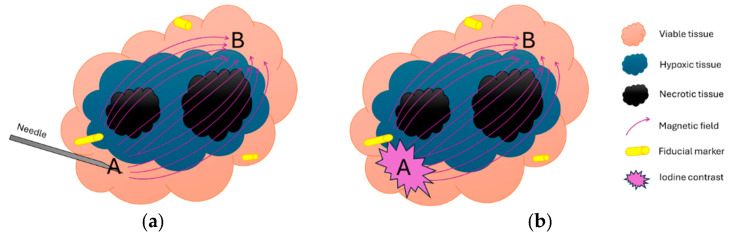
The two CBCT-based registration strategies for localizing the injection point relative to implanted gold fiducials and a target point B for bacteria guidance. (**a**) Surrogate needle registration (transabdominal/endorectal US route): The needle is advanced toward the fiducial group and imaged on CBCT with tissues in their native, undeformed position; the needle tip serves as a surrogate for injection point A. (**b**) Direct contrast bolus registration (EUS route): A small volume of iodine contrast added to the injectate is visualized directly on post-injection CBCT after endoscope removal, allowing registration of the bolus position relative to the fiducials once tissues have resumed their undeformed state.

One challenge to address is tissue deformation during EUS/US intervention. For surface lesions, when the needle insertion is guided using surface US, the US probe and its applied pressure can be removed, leaving the needle in place and the tissues in their original shape (Figure 4a). Then, the needle can be imaged using CBCT with the anatomy and tumor undeformed. This allows the needle tip to be used as a surrogate for the injection point-A in the MTBT registration step. For EUS, the presence of the endoscope deforms the internal tissues compared to the planning CT performed without the endoscope. Solutions such as electromagnetic navigation for colonoscopy exist [28] but not for the pancreas [29,30]. Emerging technologies like augmented fluoroscopy are commercially available for lung procedures using bronchoscopy but not for the pancreas [31] nor the colon. Consequently, in general, when using EUS, the soft gastrointestinal tissues that are deformed by the presence of the EUS scope cannot be fused with planning imaging, i.e., the interventional CBCT will not show the same tumor position during and after EUS removal as tissues will resume their original position [32]. At present, there are no clinical solutions to position a needle in the digestive system relative to an external treatment apparatus using EUS [29,32]. To solve this issue, we propose a second route of registration (Figure 3) that keeps the tissues in the same position as in the planning reference image. In this solution, we add a small amount of iodine contrast to the injectate and use CBCT (Figure 4b) after endoscope removal to position the injected bolus.

Lastly, in MTBT, there is a magnetic guiding apparatus, the PolarTrak, that surrounds the patient’s body. The feasibility of using this system for EUS interventions has never been tested before. While it allows some space to access the patient from both sides, it is not straightforward to conclude that EUS is a feasible technique in this environment. Clinical EUS is usually done bedside, with the patient in the left lateral decubitus position, allowing freedom of movement for the endoscopist and easy access to the patient for other providers, such as the anesthetist, nurses and technologists. In the case of MTBT and the PolarTrak, the line of sight to the monitors and freedom of scope movement need to be evaluated for the complex EUS navigation movements in the GI tract.

### 1.3. Registration of the Third Body

**Figure 5:**
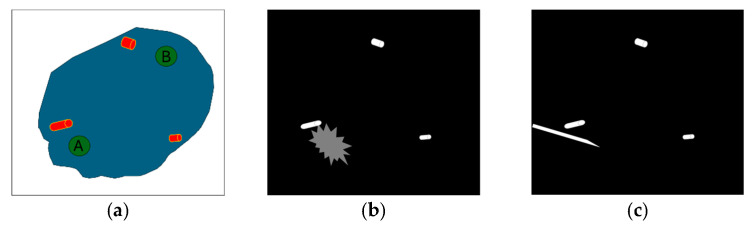
From RTStruct DICOM, guidance planning to bolus (**b**) and needle CBCT (**c**) registration. (**a**) In dark blue is the contour of a segmented tumor from a radiotherapy planification scan DICOM file showing the fiducials in red and the planned guidance points from injection (A) to target (B). (**b**) Illustration of a CBCT slice showing the same fiducials and an injected bolus at point A. (**c**) Alternative needle visualization on a CBCT slice showing the same fiducials and needle tip at injection point A.

To magnetically guide bacteria inside a tumor, we need to know exactly where the injection point (A) and the target point (B) are, and how they relate to the external magnetic field (see Figure 1). We refer to this as a “three-body registration”, linking the tumor, the injected bacteria, and the magnetic field. This adds an extra component compared to standard IGRT, allowing precise movement from point A to point B within the tumor. The full technical details of the guidance system are beyond the scope of this report. However, it is worth noting that the system builds on the same software framework used in IGRT. Like IGRT, it uses the standard radiotherapy data format Digital Imaging and Communications in Medicine (RTStruct DICOM), which includes the contours of the tumor and is linked to planning images like MRI or CT [6]. On the day of treatment, a CBCT scan is used to align the system with the patient’s current anatomy. Registration is then performed using one of the two approaches shown in Figure 3. Figure 5a shows a representation of the RTStruct file with contours of the tumor and fiducials from the planning phase, which are aligned with either a contrast injection (Figure 5b) or the needle tip position (Figure 5c).

The combined use of fiducials and contrast in the context of intratumoral injection alongside MTBT has not been addressed until now. This paper aims to assess the associated technical challenges and to facilitate the translation to clinical trials of the combined usage of implanted fiducials, US/EUS needle navigation, and CBCT registration with planning MRI/CT in the context of MTBT.

### 1.4. Note on Intratumoral Contrast Medium

Iodine contrast media are approved for oral, intra-arterial/venous, intracavity, intrathecal or intraspinal injection. To this day, there is no commercially available X-ray contrast medium approved for CBCT imaging that can be used for intratissue or intratumoral injection. We found only one study using that method [33] and one clinical trial using the iodine-based PV-10 drug with a 10% iodine *v/v* concentration [34] that showed good visibility and image confirmation at the time of injection [35]. It is known that adverse events for iodine contrast media, when they occur, are usually mild and are mostly seen with significant amounts of injected concentrated contrast [36,37] and that the clinical pharmacology, i.e., the metabolization of the iodine contrast when injected intravenously, intra-arterially or otherwise involves exiting the circulatory system or cavity to be distributed in the tissue extracellular fluid compartment (EFC) and is finally excreted unchanged by glomerular filtration [38]. As such, one can hypothesize that direct injection of low concentrations (5–10%) of iodine contrast in the tissue EFC may cause low-risk adverse events and that it poses an acceptable ethical toxicity risk–benefit profile in the context of clinical trial documentation [10,39]. This research proposes to identify the minimum amount of iodine contrast to be added to the injectate to register both the tumor (gold fiducials) and the injected bolus to the treatment apparatus (EUS-guided route in Figure 3).

### 1.5. Subregional Needle Placement in Tumor Using US/EUS Navigation and Fiducials

Tumor shape, extent, borders and subregions are not always easy to identify on US/EUS. This limits the ability of these techniques alone to guide the needle toward a planned injection site. This is where fiducial markers can play a second role in the intervention. Being able to see the fiducial markers on the US image can give an idea of where to navigate the needle when the tumor target subregion is not identifiable. Unfortunately, even if the fiducial markers can be visualized with US/EUS, they are not easily identifiable, and a combination of CBCT and US/EUS may be needed to increase confidence in adequate needle positioning during the intervention. In this study, we relied solely on gold-fiducial shine-through artifacts on US/EUS and 3D CBCT to navigate the needle toward a target defined as the center of the pre-implanted fiducial markers.

Conventional intratumoral therapy relies on passive diffusion and convection from the injection site, which produces uneven intratumoral drug distribution, limited penetration beyond the needle track, and frequent backflow or leakage into adjacent tissue; poorly perfused hypoxic and necrotic subregions, often the most treatment-resistant, are especially difficult to reach, so only a fraction of the tumor volume typically receives a therapeutic dose. MTBT is designed to overcome these limitations by adding active, magnetically directed transport: after injection, drug-loaded magnetotactic bacteria are steered along controlled field lines from the injection point (A) toward defined targets (B), including hypoxic regions to which the bacteria are naturally attracted, thereby extending coverage beyond the passive injection footprint. Establishing and quantifying the starting point of this active distribution is the specific problem addressed in this work.

The role of endoscopy in cancer management has expanded well beyond diagnosis and palliation, with EUS-guided locoregional tumor-directed therapy now representing a growing platform. Intratumoral modalities investigated via EUS in pancreatic cancer include radiofrequency ablation, ethanol ablation, injection of chemotherapeutic agents, oncolytic viruses and immunotherapeutics, brachytherapy, and, most recently, interstitial alpha radiotherapy [40,41,42,43]. These approaches collectively establish the procedural feasibility and safety of EUS-guided intratumoral access in the pancreas. MTBT extends this paradigm from passive or ablative local therapy toward actively navigated, magnetically directed intratumoral drug distribution, with the added biological advantage that magnetotactic bacteria are naturally attracted to the hypoxic and anoxic tumor microenvironment, precisely the subregions most resistant to conventional therapy and least accessible to passive delivery.

This introduction establishes the feasibility of the concept of precise intratumoral injection using a combination of implanted fiducials, EUS-guided needle navigation, iodine contrast-enhanced injectate, fiducial shine-through, and CBCT-based registration, despite the associated MTBT constraints. In this preclinical study, we investigated strategies to optimize and validate key parameters, including contrast concentration, the spatial constraints of EUS guidance in the treatment apparatus, and the accuracy of three-body image registration.

## 2. Materials and Methods

### 2.1. Animal Preparation and Initial Setup

Three healthy domestic female swine (~25 kg, approximately 3–4 months old) were used, obtained from an authorized/certified supplier. All procedures were performed under an approved institutional animal ethics protocol (2I21010GSp; approved 22 April 2021) and conducted according to the ARRIVE guidelines 2.0 Essential 10 criteria, as detailed in the Supplementary Materials Table S1. Animals were prepared for pancreatic or rectal intervention with general anesthesia and peri-procedural antibiotics; an enema was administered before rectal procedures. They were positioned either prone for EUS or supine for abdominal US and endorectal US in an interventional angiography suite and placed on a 3D robotic operating table. Initial reference imaging was performed using a Siemens Artis CBCT system (CBCT Artis Q system Siemens Healthcare GmbH, Forchheim, Germany) to obtain baseline anatomical details. Three fiducials (1.0 mm × 3.0 mm Best Medical International, Inc., Springfield, VA, USA) were implanted in the pancreas and rectum. Fiducial implantation was conducted via two different approaches depending on the animal. EUS (GF-UCT180 linear array echoendoscope; Olympus Medical Systems Corporation, Tokyo, Japan) was used for two animals, and an abdominal surface probe and endorectal probe US (Flex focus 400 8820, 8848 BK Medical, Burlington, MA, USA) were employed for one animal. The choice of guidance modality was assigned by operator specialty (EUS by a gastroenterologist; transabdominal and endorectal US by an interventional radiologist) rather than by animal-specific factors. The two approaches were assessed independently for feasibility of the same workflow; no head-to-head comparison between EUS and US was performed or intended, given the pilot sample size.

**Figure 6:**
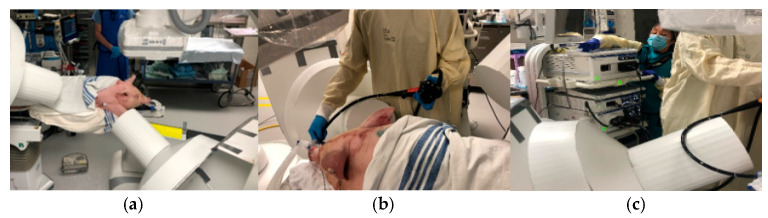
Cardboard replica of the PolarTrak on one side of the operating table (**a**); endoscopist access and freedom of movement (**b**); limited access for the technologist (**c**).

To evaluate the feasibility of the EUS procedure, a cardboard replica of the PolarTrak structure was positioned on the endoscopist side of the table to simulate real clinical spatial constraints on the operator’s movements (see Figure 6). A follow-up CBCT was performed immediately after the fiducial placement to verify their positions and ensure accurate localization.

### 2.2. Needle Navigation and Injection

**Figure 7:**
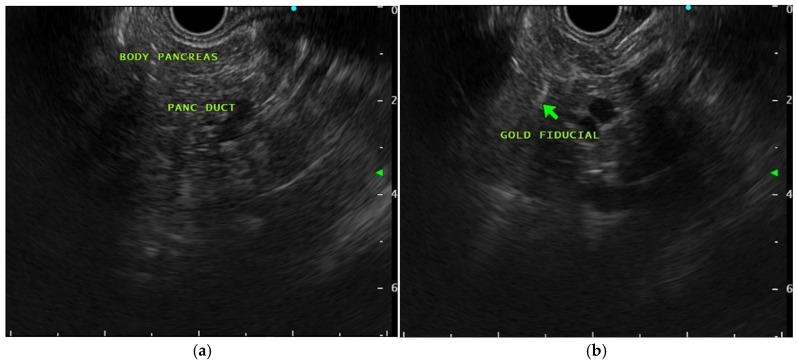
Animal pancreas EUS structure identification prior to and after fiducial implantation. Image (**a**) shows the pancreas regions clearly identified under EUS navigation. Image (**b**) shows a shine-through of the gold fiducials that helped the interventionist/endoscopist to find the injection sites.

Needle navigation was performed using the same imaging guidance technique as for fiducial implantation. Efforts were made to identify fiducial shine-through on the US image to optimize the needle trajectory toward the center of the fiducial group. Imaging with CBCT was again utilized to confirm the needle’s position during abdominal US-guided navigation (Figure 7). Briefly, 2 mL of saline solution mixed with Isovue 370 contrast agent at varying concentrations (15%, 10%, and 5%, one for each animal) was injected into the targeted area. Post-injection CBCT imaging was conducted to visualize the distribution and location of the injected bolus. A blinded 5-point Likert scale was used by an expert radiologist to qualitatively assess both the visibility and the presence of imaging artifacts associated with the injected contrast agent and gold seeds. Visibility was rated from 1 (not visible) to 5 (excellent visibility), while artifact severity was rated from 1 (severe artifact) to 5 (no artifact). This semi-quantitative scoring method provides a standardized and reproducible evaluation of image quality from a clinical perspective.

**Figure 8:**
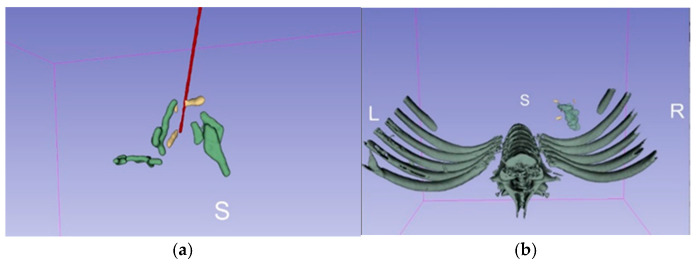
Segmented CBCT images of the two experiments variation. (**a**) Abdominal US with needle (in red) and (**b**) EUS without needle. Fiducials are yellow, and the injected bolus is green. Image (**a**) shows that injection can result in a dispersed bolus.

Image segmentation was conducted using the 3D Slicer software 4.11.20210226 platform (Figure 8), applying intensity thresholding techniques to the CBCT datasets to delineate the fiducial markers, the needle trajectory, and the injected contrast bolus. Each of these structures was segmented in three dimensions based on its intensity profile. Following segmentation, the geometric centroids of the fiducials, needle tip, and bolus were automatically computed. The spatial relationships between these key anatomical and procedural landmarks were then quantified by calculating the distances between their respective centroids, enabling an objective assessment of targeting accuracy.

## 3. Results

We succeeded in implanting fiducials and injecting contrast in a total of three pancreas sites and one rectal site using EUS in two animals, and one pancreas site and one rectum site using abdominal and endorectal US probes in one animal. The first animal received one injection in the pancreas, the second animal received two injections (one in the pancreas and one in the rectum), and the third animal received two injections in the pancreas (body and tail) and one in the rectum, as we improved our techniques and were able to perform more procedures on the same day.

With the PolarTrak cardboard simulator in place, both the US and EUS approaches were successfully performed with minimal interference. Some EUS movements by the endoscopist required the 3D table to be moved inferior to the animal when accessing via the mouth and moved back to the middle of the system for CBCT scans. Access to the EUS instrument cart for the medical technologist was constrained when interaction with the front of the cart was required. A reasonably adequate position of the cart was found by extending the reach of the monitor using the cart extension arm.

**Figure 9:**
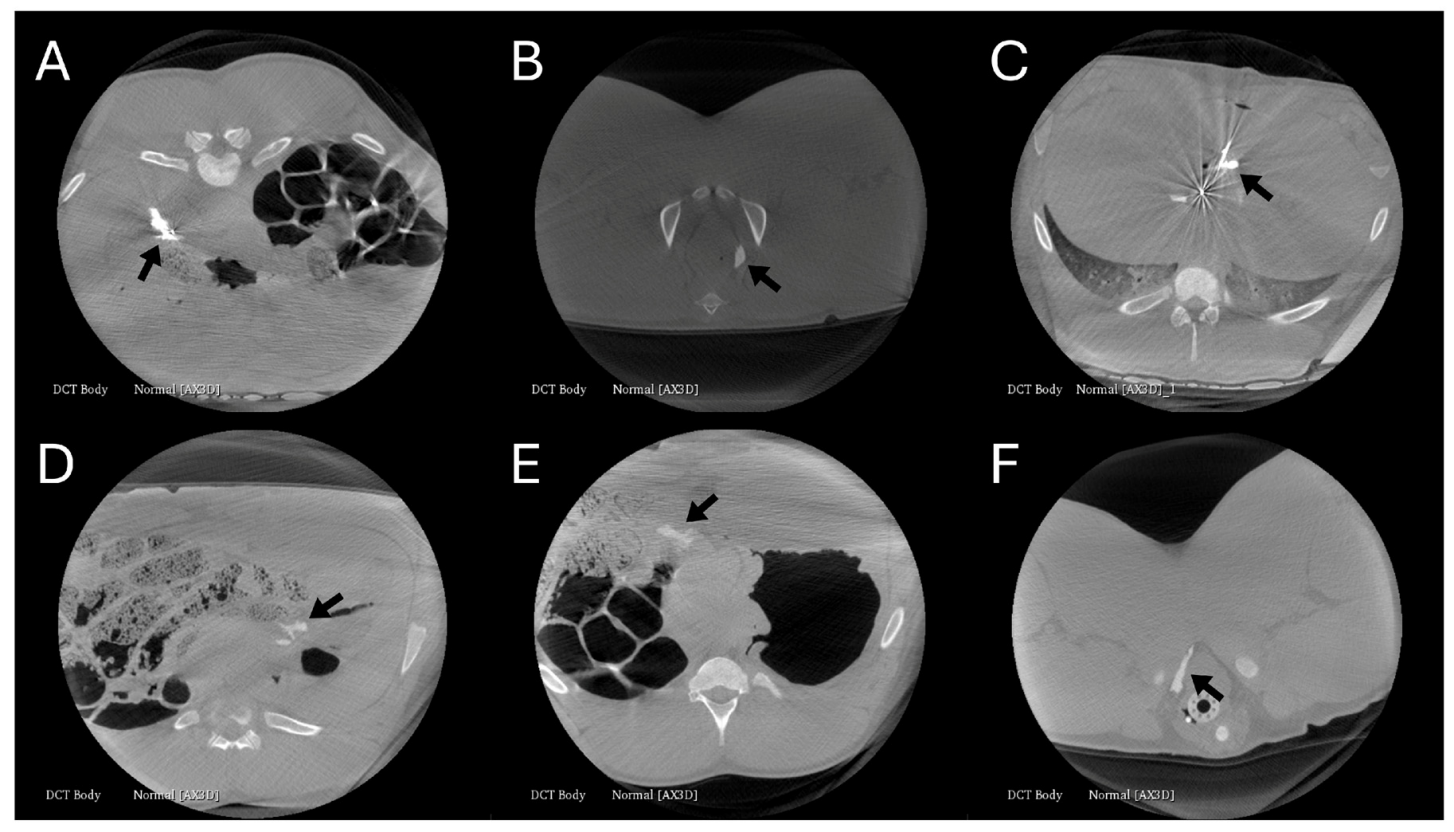
Arrows point at the injected contrast bolus showing relative visibility on CBCT. Insert (**A**) is 15% *v/v* (55.5 mg/mL), (**B**,**C**) are 10% *v/v* (37 mg/mL), and (**D**–**F**) are 5% *v/v* (18.5 mg/mL) Isovue-370 concentrations.

**Figure 10:**
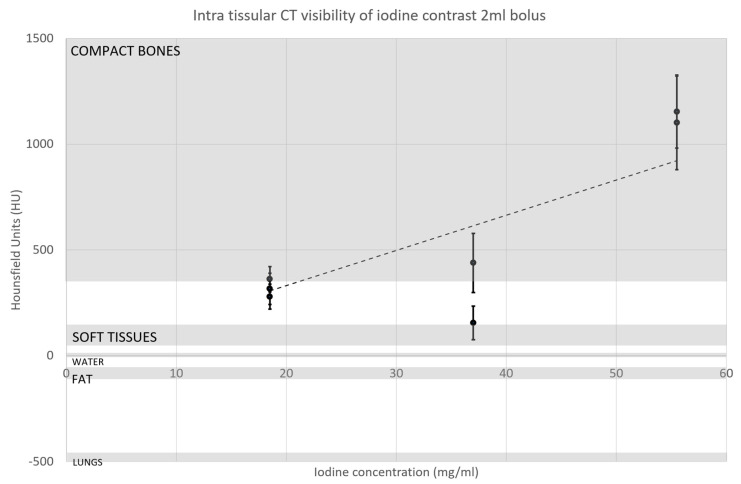
Density of the ISOVUE-370 contrast bolus at three concentrations (18.5, 37, and 55.5 mg/mL) relative to the density ranges of representative tissues (lung, fat, water, soft tissue, and compact bone).

When the injected bolus was fully contained in the organs, it showed a relatively good agreement between the actual liquid volume and the CBCT segmented image volume, i.e., 2 mL injected gives approximately 2000 mm^3^. Table 1, Figure 9 and Figure 10 summarize the contrast measurements, radiologist assessment, typical bolus shape, and comparison with reference tissue values. A 5% *v/v* Isovue-370 concentration was sufficient to fall in the Hounsfield value range between soft tissues and compact bone and to be clearly identified by the radiologist.

Using EUS or US and shine-through fiducial targeting, the distance between the injected bolus and needle positioning relative to the fiducial centroids was below 2 cm in five sites and 8.2 cm in one site due to injection leakage into a body cavity (Table 1, distance), showing an overall good targeting precision with both approaches when the injection is in the stroma of the tissue.

## 4. Discussion

In this preclinical study, we describe the technique for EUS-/US-guided needle navigation for GI and pancreatic cancer MTBT for the first time. We demonstrated that the registration of a tumor and a localized injectate is possible with two different techniques with a clinically relevant localization accuracy on the order of 2 cm for defining the initial injection point without an actual real tumor mass. We propose methods that rely on state-of-the-art IGRT image registration protocols to which we added interventional EUS/US needle navigation using fiducial shine-through. To this end, we assessed an interventional technique mimicking intratumoral injection in healthy swine using either a contrast bolus or the needle tip and interventional CBCT-to-planning CT registration, enabling localization of the injectate in a tumor subregional zone that we named three-body registration. This enhanced registration is required in the case of MTBT to actively distribute the drug in the tumor after the initial passive injected distribution. These in vivo experiments provide confidence that the two different methods, needle tip and iodine contrast bolus, can be used to identify the starting point for MTBT on medical imaging, thus allowing for 3D bacteria navigation.

The novelty of this approach does not reside solely in the use of US-guided injection or iodinated contrast, which are established interventional tools. Rather, the contribution lies in adapting these tools to the specific requirements of MTBT, where the injected bolus is not the final therapeutic distribution but the starting point for subsequent magnetic navigation. Therefore, the injection site must be registered not only relative to the tumor anatomy, but also relative to the magnetic guidance apparatus. This three-body registration is specific to MTBT and differs from conventional intratumoral injection of passive agents.

We proposed adding iodine contrast to the injected solution to localize the bacteria-drug complex on CBCT. We assumed that the contrast would not negatively interact with the drug or bacteria and that the contrast would represent the initial position of the bacteria. Of note, the actual production of the bacteria–drug complex does not include iodine contrast in its formulation, though it cannot be visualized on interventional CBCT. We must also assume that the bacteria will not separate from the injected liquid by tissue barrier filtration. This may not be a valid assumption in healthy tissue in which the network of cells is well-organized and tightly packed, and the space for fluid movement is on the order of nanometers. In tumors, however, there is more space between disorganized cancer cells. Indeed, previous intratumoral in vitro and in vivo work showed that the bacteria can distribute in such an environment [1,44] and that the iodine/bacteria distribution superposition assumption at the time of injection is reasonable for tumors.

We demonstrate that the combined use of CBCT and EUS gold fiducial shine-through allows the endoscopist to navigate a needle for intratumoral injection in the GI tract (rectum) and pancreas of swine. This technique can be extrapolated to other GI tumor sites, including the esophagus, stomach, duodenum, and colon. In the absence of a real tumor, using only medical CBCT information and the shine-through of the fiducial, we found that the endoscopist was more confident in needle positioning. This should also help in real cases in which a distinct lesion can be seen on both planning imaging with fiducials and on EUS.

Adding iodine contrast to the injectate is a trade-off between therapeutic and visualization efficacy, as contrast occupies some percentage of the total allowed amount of liquid injected. The limit is also a function of the dilution of the injectate in the tissues. We found that about the same volume of injectate was visible on CBCT, i.e., 1 mL injected yielded a 1 cm^3^ bolus on the image (a 1:1 ratio). This indicates the need to optimize the amount of iodine for a 1:1 ratio and aim to be in the contrast zone between bone and soft tissues. Using the highest concentration of iodine per mL available on the market, Isovue-370, we found that a 5% *v/v* concentration was needed to reach the 300 Hounsfield unit range, assuming a 1:1 injection ratio. This may need to be adjusted if, in tumors, the 1:1 ratio is not maintained. For example, if the injection does not spread as much in a tumor because of increased cellularity or interstitial pressure, this 5% may be reduced. Alternatively, if it is found that the injectate spreads more within a tumor because of large necrotic zones, this 5% may need to be increased to ensure good contrast on CBCT.

A few outstanding challenges are worth noting. We assumed that either the needle tip or the contrast bolus represents the real initial bacteria position. When using the needle tip registration only, the injectate does not necessarily follow a smooth spherical distribution at the tip end. In one case, we found that the injected fluid accumulated in nearby cavities (Figure 8) and spread in a non-uniform fashion from the end of the needle tip. This indicates that using the needle tip alone gives only a rough idea of the initial injection position and distribution. While imprecise, this method gives at least a general idea of the injection point for the MTBT starting point.

In our protocol, the time between contrast injection and CBCT was short, i.e., less than 5 min. At one point, we performed two CBCT scans 5 min and 10 min apart and observed that the contrast was almost gone after 10 min. This implies that the time between injection and imaging may be a sensitive parameter. This concern was noted in healthy, well-perfused organs, whereas the injectate likely behaves differently in tumors. This should be further assessed in clinical cases, and alternative strategies should be employed if the maximum time to CBCT proves too short given fast contrast wash-out.

One study objective was to evaluate the PolarTrak physical constraints for EUS-guided GI/pancreas intervention. The presence of equipment around the patient did somewhat limit access to the patient’s mouth and constrain endoscopist movements. We found it useful to move the patient’s head inferiorly to allow for easier mouth access without being blocked by the bottom magnetic head, and we expect the PolarTrak’s robotic table to assist in this regard. We also found that the technologist had limited access to the EUS cart. This problem may be addressed by using a second monitor for the endoscopist, allowing for repositioning of the EUS cart for better technologist access.

A 2 cm localization tolerance may be considered large relative to some tumor subregions, and we address this directly. Unlike ablative therapies, in which the delivered agent or energy must itself cover the target, MTBT uses the injection point only as the origin for subsequent magnetically guided transport, so the acceptable initial localization error is coupled to the magnetic navigation range rather than to the lesion margin alone. Moreover, the reported values are centroid-to-centroid distances obtained in healthy tissue without a target lesion, and the single 8.2 cm value reflected injectate leakage into a cavity rather than a navigation failure. For reference, fiducial-based IGRT operates with planning margins on the order of millimeters to approximately 1 cm, and EUS-guided interventions routinely target lesions of comparable size; the present accuracy of about 2 cm in a lesion-free model is therefore a reasonable proof of concept but should not be interpreted as a validated clinical threshold. Clinically acceptable accuracy for MTBT should be defined prospectively relative to tumor size, subregional targets, and the effective magnetic navigation distance, and confirmed in tumor-bearing models.

This study has a few limitations. First, the experiments were performed in only three animals, which restricts the generalizability of the findings. Second, each imaging modality was operated by a single specialist, which may have introduced operator-dependent variability and limited the assessment of reproducibility across different users. Third, only one type of contrast medium was tested, leaving unanswered the question of whether alternative agents could improve detectability and registration accuracy. These limitations highlight the need for further validation in larger cohorts, with multiple operators and a broader range of contrast conditions. This proof-of-concept study was conducted in three animals in accordance with 3R principles; the small sample size precludes statistical inference and limits generalizability. The findings are intended to establish technical feasibility and define the procedural parameters needed to inform first-in-human clinical trial design. The key assumptions of this work, namely, that iodine contrast approximates the initial bacterial distribution, that bolus geometry persists over the imaging window, and that healthy tissue behavior extrapolates to tumors, remain to be validated: contrast is used only as a surrogate for the injection origin because the clinical bacteria–drug formulation contains no iodine; rapid contrast wash-out was observed in well-perfused organs (motivating CBCT acquisition within a few minutes of injection); and the injection-to-image volume relationship may differ in the larger interstitial spaces of tumors.

## 5. Conclusions

The present study demonstrates the feasibility of an EUS/US-CBCT workflow designed to localize the initial injection site required for subsequent magnetotactic bacteria navigation. The essential three-body registration needed for MTBT was successfully demonstrated using gold fiducials combined with either the needle tip or iodine contrast and was achieved with precision; the optimal iodine contrast concentration for interventional CBCT was identified, all within the spatial constraints of the designated PolarTrak navigation device. This accuracy should not be interpreted as resolving microscopic tumor transport properties. Rather, it defines the achievable clinical localization of the initial bacterial bolus. These findings encourage and inform the use of this promising novel cancer treatment in upcoming clinical studies.

## Figures and Tables

**Table 1:** Hounsfield values for implanted gold fiducials and 2 mL injected iodine contrast bolus.

Site/ Structure	Volume (mm^3^)	Radiodensity (Hounsfield)	Distance (mm)	CBCT ^1^ Visibility	CBCT ^1^ Artifact	FL ^2^ Visibility	FL ^2^ Artifact
Pancreas 1							
Gold Seed	40	4853		5	3.5	5	1
Contrast 15%	963	1154	9	5	1	N/A	N/A
Pancreas 2							
Gold Seed	159	3473		5	3	5	1
Contrast 10%	1267	156	14	5	1	N/A	N/A
Pancreas 3							
Gold Seed	43	4788		5	4	5	1
Contrast 5%	1242	317	6	3	1	N/A	N/A
Pancreas 4							
Gold Seed	16	4006		5	4	5	1
Contrast 5%	3071	280	16	3	1	N/A	N/A
Rectum 1							
Gold Seed	46	3645		5	3.5	5	1
Contrast 10%	2033	439	3	5	1	N/A	N/A
Rectum 2							
Gold Seed	13	4816		5	4	5	1
Contrast 5%	1592	362	82	3	1	N/A	N/A

^1^ CBCT: cone-beam computed tomography, ^2^ FL: fluoroscopy. N/A: Not Applicable, because fluoroscopy is not used for contrast bolus visualization.

## Data Availability

The data presented in this study are available upon request from the corresponding author.

## Supplementary Materials

The following supporting information can be downloaded at: https://www.mdpi.com/article/10.3390/cancers18152427/s1, Table S1: The ARRIVE guidelines 2.0: Author checklist [45].
