# Supplementary material for: Preclinical Optimization of Magnetotactic Bacteria Therapy for the Treatment of Pancreatic and Rectal Cancer
Source: Cancers (Basel). 2026 Jul 28;18(15):2427. doi: 10.3390/cancers18152427 (PMC13465606; doi:10.3390/cancers18152427)
Supplement: Supplementary file 1 [file cancers-18-02427-s001.zip › cancers-4391781-supplementary.pdf]

Table S1. The ARRIVE guidelines 2.0: Author checklist [45].

The ARRIVE Essential 10				
These items are the basic minimum to include in a manuscript. Without this information, readers and reviewers cannot assess the reliability of the findings.				
**Item**			**Recommendation**	**Section/line number, or reason for not reporting**
**Study design**	1		For each experiment, provide brief details of study design including:	Exploratory pre-study with limited number of experimental units
		a.	The groups being compared, including control groups. If no control group has been used, the rationale should be stated.	
		b.	The experimental unit (e.g. a single animal, litter, or cage of animals).	Single animal
**Sample size**	2	a.	Specify the exact number of experimental units allocated to each group, and the total number in each experiment. Also indicate the total number of animals used.	3
		b.	Explain how the sample size was decided. Provide details of any *a priori* sample size calculation, if done.	Exploratory proof-of-concept with limited number of experimental units
**Inclusion and exclusion criteria**	3	a.	Describe any criteria used for including and excluding animals (or experimental units) during the experiment, and data points during the analysis. Specify if these criteria were established *a priori.* If no criteria were set, state this explicitly.	Healthy animal
		b.	For each experimental group, report any animals, experimental units or data points not included in the analysis and explain why. If there were no exclusions, state so.	No exclusion
		c.	For each analysis, report the exact value of *n* in each experimental group.	2 anatomical sites, rectum and pancreas
**Randomization**	4	a.	State whether randomization was used to allocate experimental units to control and treatment groups. If done, provide the method used to generate the randomization sequence.	No randomization
		b.	Describe the strategy used to minimize potential confounders such as the order of treatments and measurements, or animal/cage location. If confounders were not controlled, state this explicitly.	No confounder controlled
**Blinding**	5		Describe who was aware of the group allocation at the different stages of the experiment (during the allocation, the conduct of the experiment, the outcome assessment, and the data analysis).	Animal facility animal care personnel and Charles Tremblay, Corey Miller, Gilles Soulez, Miriam Santos
**Outcome measures**	6	a.	Clearly define all outcome measures assessed (e.g. cell death, molecular markers, or behavioural changes).	US and CBCT imaging outcomes
		b.	For hypothesis-testing studies, specify the primary outcome measure, i.e. the outcome measure that was used to determine the sample size.	Primary outcome measures are pre-stated in the Methods section rather than appearing only in the Results section
**Statistical methods**	7	a.	Provide details of the statistical methods used for each analysis, including software used.	Not applicable to this small cohort

		b. Describe any methods used to assess whether the data met the assumptions of the statistical approach, and what was done if the assumptions were not met.	Not applicable
**Experimental animals**	8	a. Provide species-appropriate details of the animals used, including species, strain and substrain, sex, age or developmental stage, and, if relevant, weight.	Domestic swine
		b. Provide further relevant information on the provenance of animals, health/immune status, genetic modification status, genotype, and any previous procedures.	Healthy, 25 kg
**Experimental procedures**	9	For each experimental group, including controls, describe the procedures in enough detail to allow others to replicate them, including:<br>a. What was done, how it was done and what was used.<br>b. When and how often.<br>c. Where (including detail of any acclimatization periods).<br>d. Why (provide rationale for procedures).	a) Anesthesia, fiducial implantation, contrast injection, ultrasound and CBCT imaging<br>b) Fiducial implantation and contrast injection performed once. Anesthesia and imaging performed twice, two weeks apart.<br>c) Centre de Recherche du Centre Hospitalier de l'Université de Montréal<br>d) Pre-clinical development of a proof-of-concept and optimization of methods as a preparation for first in human trial
**Results**	10	For each experiment conducted, including independent replications, report:<br>a. Summary/descriptive statistics for each experimental group, with a measure of variability where applicable (e.g. mean and SD, or median and range).<br>b. If applicable, the effect size with a confidence interval.	Not applicable due to the small cohort<br>Not applicable
